# Supplementary material for: Graphene-Based Coating to Mitigate Biofilm Development in Marine Environments
Source: Nanomaterials (Basel). 2023 Jan 18;13(3):381. doi: 10.3390/nano13030381 (PMC9919625; doi:10.3390/nano13030381)
Supplement: Supplementary file 1 [file nanomaterials-13-00381-s001.zip › nanomaterials-2144708-supplementary.pdf]

Supplementary Material

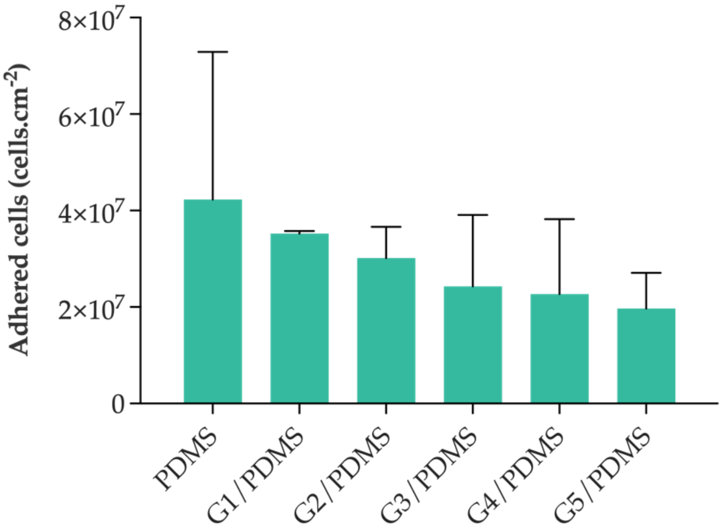

Figure S1. *C. marina* adhered cells on PDMS, G1/PDMS, G2/PDMS, G3/PDMS, G4/PDMS, and G5/PDMS surfaces after incubation for 7.5 h at 185 rpm. Results are presented as mean ± SD.

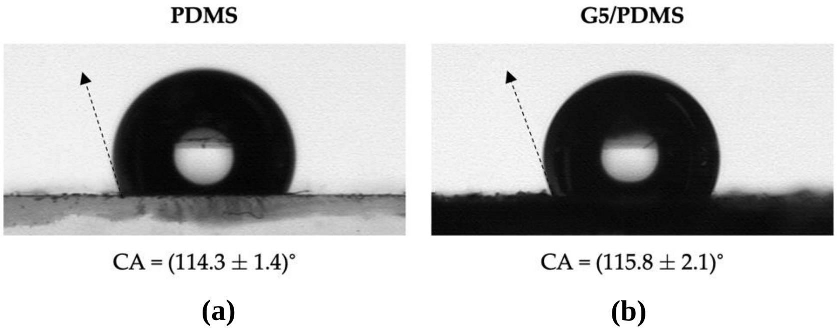

Figure S2. Representative images of water contact angle (CA) measurements on PDMS (**a**) and G5/PDMS (**b**) surfaces.

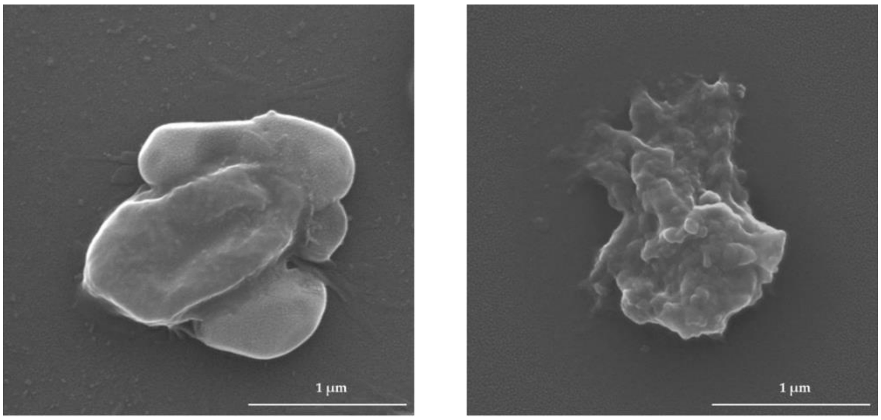

(a) (b)

Figure S3. Scanning Electron Microscopy (SEM) of *C. marina* cells not exposed (**a**) and exposed to GNP 5% (w/v) (**b**) for 24 h (magnification of 50,000× and white scale bars of 1 μm). Cell damage occurred in bacterial cells exposed to graphene.

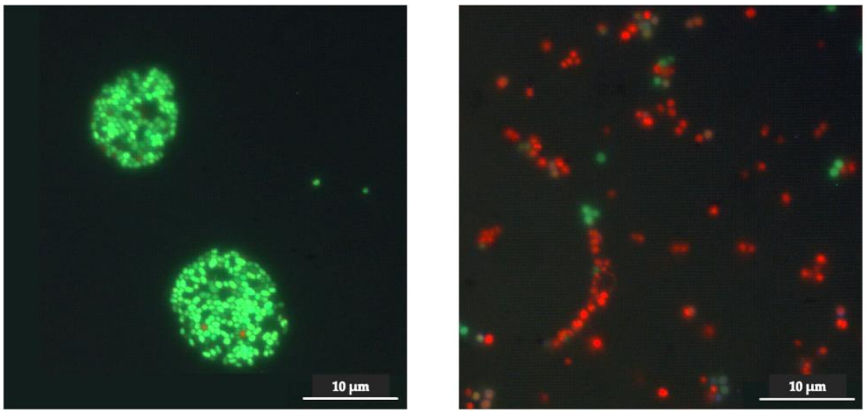

(a) (b)

Figure S4. Epifluorescence microscopy visualization of *C. marina* cells not exposed (**a**) and exposed to GNP 5% (w/v) (**b**) for 24 h (scale bars of 10 μm). Viable cells appear green and dead cells appear red after being stained with the Invitrogen Live/Dead® kit.
